# Sitting Postural Management to Prevent Migration Percentage Progression in Non-Ambulatory Children with Cerebral Palsy: Randomized Controlled Trial Preliminary Data

**DOI:** 10.3390/jcm13113129

**Published:** 2024-05-27

**Authors:** Silvia Faccioli, Irene Maggi, Emanuela Pagliano, Claudia Migliorini, Arianna Michelutti, Liliana Guerra, Anna Ronchetti, Giovanna Cristella, Nicoletta Battisti, Lara Mancini, Odoardo Picciolini, Silvia Alboresi, Antonio Trabacca, Shaniko Kaleci

**Affiliations:** 1 Pediatric Rehabilitation Unit, Azienda Unità Sanitaria Locale IRCCS of Reggio Emilia, 42122 Reggio Emilia, Italy; irene.maggi@ausl.re.it (I.M.); silvia.alboresi@ausl.re.it (S.A.); 2 PhD Program in Clinical and Experimental Medicine, Department of Biomedical, Metabolic and Neural Sciences, University of Modena and Reggio Emilia, 41125 Modena, Italy; 3 Neurodevelopmental Unit, Fondazione IRCCS Istituto Neurologico Carlo Besta, 20133 Milan, Italy; emanuela.pagliano@istituto-besta.it; 4 Rehabilitation Department, AULSS 9 Scaligera, 37122 Verona, Italy; claudia.migliorini@aulss9.veneto.it; 5 Scientific Institute IRCCS E. Medea, 33037 Pasian di Prato (UD), Italy; arianna.michelutti@lanostrafamiglia.it; 6 Pediatric Neuropsychiatric Unit, Azienda Unità Sanitaria Locale Modena, 41122 Modena, Italy; l.guerra@ausl.mo.it; 7 Physical Medicine and Rehabilitation Unit, IRCCS Istituto Giannina Gaslini, 16147 Genoa, Italy; annaronchetti@gaslini.org; 8 IRCCS Don Carlo Gnocchi Foundation, 50143 Florence, Italy; gcristella@dongnocchi.it; 9 Pediatric Physical Medicine and Rehabilitation Unit, IRCCS Institute of Neurological Sciences, 40124 Bologna, Italy; nicoletta.battisti@isnb.it; 10 Physical Medicine and Rehabilitation Unit, Santa Maria delle Croci Hospital, Azienda Unità Sanitaria Locale Romagna, 48100 Ravenna, Italy; lara.mancini@auslromagna.it; 11 Pediatric Physical Medicine and Rehabilitation Unit, IRCCS Ca’ Granda Ospedale Maggiore Polyclinic Hospital, 20122 Milan, Italy; odoardo.picciolini@policlinico.mi.it; 12 Unit for Severe Disabilities in Developmental Age and Young Adults, Scientific Institute IRCCS E. Medea, 72100 Brindisi, Italy; antonio.trabacca@lanostrafamiglia.it; 13 Surgical Medical and Dental Department of Morphological Sciences Related to Transplant, Oncology and Regenerative Medicine, University of Modena and Reggio Emilia, 41125 Modena, Italy; shaniko.kaleci@unimore.it

**Keywords:** self-help devices, hip dislocation, botulinum toxins, rehabilitation, posture, physical therapy, hip surveillance

## Abstract

**Background/Objectives**: To determine whether a sitting position with the femoral heads centered into the acetabulum is more effective than the usual sitting position in preventing migration percentage progression in non-ambulatory children with bilateral cerebral palsy. **Methods**: This was a multicenter, randomized controlled trial. Inclusion criteria: spastic or dyskinetic cerebral palsy, Gross Motor Function Classification System level IV-V, age 1–6 years, migration percentage <41%, and informed consent. Exclusion criteria: contractures affecting the hip, anterior luxation, previous hip surgery, and lumbar scoliosis. The treatment group sat with their hips significantly abducted to reduce the head into the acetabulum in a customized system for at least five hours/day for two years. Controls sat with the pelvis and lower limbs aligned but the hips less abducted in an adaptive seating system. The primary outcome was migration percentage (MP) progression. Health-related quality of life and family satisfaction were among the secondary outcomes. The study was approved by the local ethics board and conducted in accordance with CONSORT reporting guidelines. ClinicalTrials.gov ID: NCT04603625. Results: Overall median MP progression was 1.6 after the first year and 2.5 after the second year. No significant differences were observed between the groups. MP exceeded 40% and 50% in 1.8% and 0% of the experimental group and 5.4% and 3.6% of controls in years 1 and 2, respectively. Both groups expressed satisfaction with the postural system and stable health-related quality of life. **Conclusions**: MP remained stable over the two-year period in both groups. Considering outliers which progressed over 50%, a more protective trend of the hip-centering sitting approach emerged, but this needs to be confirmed in a final, larger dataset.

## 1. Introduction

Children affected by cerebral palsy (CP) are at an increased risk of progressive hip luxation. Complete luxation, also called dislocation, corresponds to a 100% migration percentage (MP); subluxation or displacement is mostly defined as an MP of between 30% and 99% [1]. The incidence of subluxation is related to CP severity in terms of gross motor skills, as defined by the Gross Motor Function Classification System (GMFCS). More severe non-ambulatory bilateral patients (GMFCS IV and V) are known to be the most vulnerable to hip subluxation [2,3]. Spastic and dyskinetic subtypes are more often involved than ataxic CP [3,4], with MP increasing mostly in early ages in spastic CP while having a more variable trend in the dyskinetic subtype [5].

The evidence supports screening programs [6,7] to implement timely management. Among the interventions described are postural management (PM) and spasticity treatment, although early surgical approaches are recommended [8,9]. Nevertheless, surgery itself has a risk of recurrence in children with GMFCS level IV-V and in surgery at a younger age [8,9]. Postural management is recommended by guidelines to prevent or delay the development of contractures or skeletal deformities in children with CP [7,10]. PM may therefore play a role in preventing an increase in MP, thus overcoming the trend of relapse after surgery. In particular, PM may contribute to keeping the MP below the point of no return (MP 46–50%), beyond which no spontaneous MP reduction can be expected and increasing hip displacement may occur unless surgery is performed [11,12]. Previous studies have investigated conservative approaches to prevent MP increase, such as standing or seating devices [13,14,15,16] or botulinum combined with abduction orthoses [17,18]. The results were inconclusive, however, given their small sample sizes and low level of evidence. A recent study by Martinsson et al. [19] demonstrated the protective role of using an abducted standing frame for 10 h/week in young subjects with GMFCS level IV-V. Nonetheless, these patients spend most of their time sitting, and, in our experience, not all of them tolerate a standing position with the support of assistive devices. Thus, the sitting position is crucial for them. Therefore, what is the best sitting position?

The aim of the present randomized controlled trial was to verify whether sitting with the femoral heads centered into the acetabulum on a customized seating system for at least five hours per day over a two-year period was more effective in preventing MP progression than an unspecific sitting position. Preliminary data are presented.

## 2. Materials and Methods

This multicenter, randomized controlled trial (CP-lux-prev) compared two different postural management approaches for the sitting position. The study was approved by the Area Vasta Emilia Nord Ethics Board on 14 July 2020 (723/2020/SPER/AUSLRE) and was registered on ClinicalTrials.gov (NCT04603625). The study was conducted in accordance with CONSORT reporting guidelines [20].

The experimental group (hip-centering sitting, HCS) sat with the hips positioned at an abduction–flexion angle to ensure centering of the femoral head. This position was determined based on a clinical examination by a physiatrist or physiotherapist according to the method described by Lespargot et al. [13,21]. More details are available in the Supplementary Data (study protocol).

**Figure 1:**
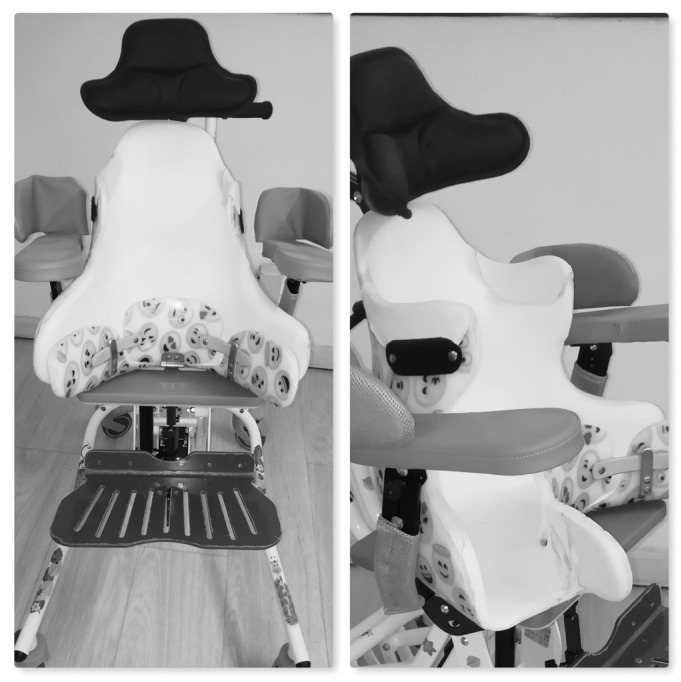
Example of the customized seating system, which maintains the femoral heads centered into the acetabulum and ensures a comfortable and safe position (HCS group).

**Figure 2:**
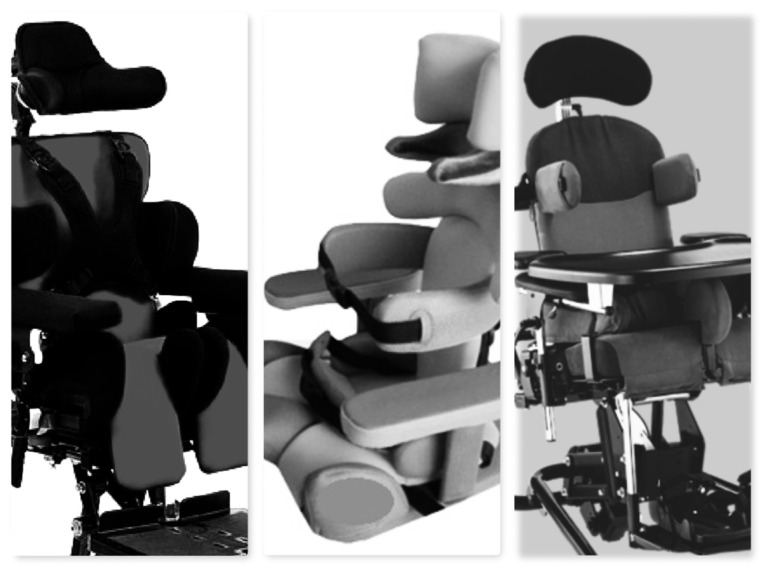
Examples of adaptive seating systems, ensuring axial alignment and a comfortable and safe position (TS group).

The desired sitting position was maintained using a customized seating system (Figure 1), similar to the previously described “siège moulé” [13,21]. The traditional treatment group (traditional sitting, TS) sat using an adaptive (Figure 2) seating system that ensures support and alignment of the trunk and pelvis but does not center the femoral head into the acetabulum.

Generally, the HCS group required significantly higher hip abduction than the TS group. In both groups, a comfortable and safe sitting position was guaranteed, and maximum attention was paid to facilitate the child’s participation in daily activities. Individual clinical features such as functional level and dystonia or hypotonia, caregivers’ needs, and contextual barriers were also considered. The seating system was placed on a wheelchair, stroller, or hi-low home base, according to family and contextual needs; a tilt-in-space base was provided for patients with a more severe condition. Both seating systems were used in a customary daily life context for at least five hours/day for 24 months (at least 12 months in these preliminary data). This cut-off was chosen according to the conditions used in a previous study by Picciolini et al. [13].

The inclusion criteria were spastic or dyskinetic CP according to the Surveillance of CP in Europe (SCPE) classification; quadriplegic patients at GMFCS level IV or V; age 1–6 years; MP < 41% measured on a radiograph acquired no more than three months prior to recruitment; and informed consent from the child’s parents or legal guardian, as per the local Ethics Board. Only patients who underwent the first year of assessment were included in the present preliminary study. The principal investigator of each trial site (experienced physiatrists and pediatric neuropsychiatrists) classified the patients according to GMFCS-E&R by Palisano et al. [22].

The exclusion criteria were hip abduction passive range of movement (pROM) <30°; knee extension pROM limitation with flexion >15° in the supine position; Thomas test >15°; anterior hip luxation; previous reconstructive surgery; soft tissue surgery in the last 12 months; and lumbar scoliosis >20° Cobb.

After enrolment, the participants were randomly assigned to the study arms. The randomization list was created via block randomization of random sizes of two, four, and six, with a 1:1 allocation ratio stratified according to participating sites to allow for competitive enrolment. The statistical software R-4.0.0 [23] was used to formulate the randomization list. Allocation was concealed through central randomization, which was performed by statisticians who had no direct contact with the clinical aspects of the trial.

**Figure 3:**
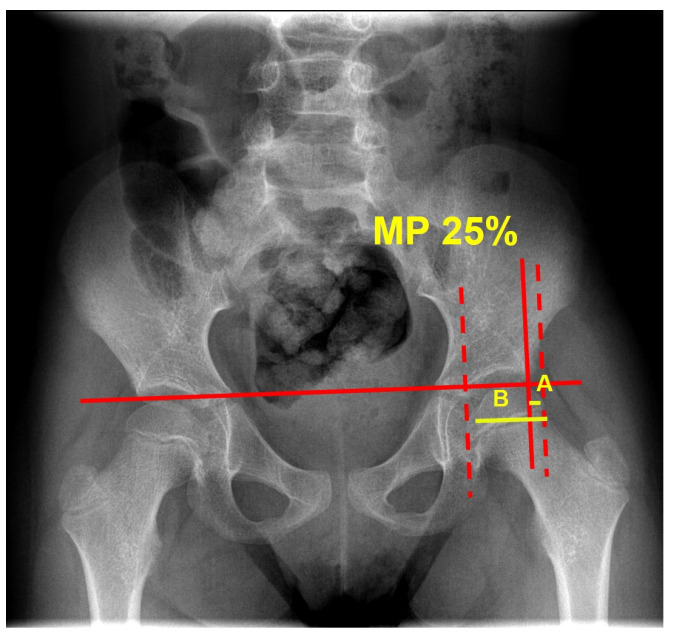
Example of an anterior–posterior radiograph of the pelvis with calculation of the migration percentage (MP): MP = A/B × 100. A represents the portion of ossified femoral head laying lateral to Perkin’s line (vertical line drawn through the lateral acetabular margin and perpendicular to Hilgenreiner’s line, which passes through the superior aspect of the triradiate cartilage). B represents the whole ossified femoral head.

The mean migration percentage progression from baseline (T0) to first- and second-year assessments (T12 and T24) was considered as the primary outcome measure. MP was assessed blinded on an anterior–posterior radiograph of the pelvis (Figure 3).

Several secondary outcomes were considered.

The percentage of hips which exceeded MP 40% was considered as a secondary outcome.

Two questionnaires (quality of life according to the Caregivers Priorities and Child Health Index of Life with Disabilities (CPCHILD) [24,25] and compliance and satisfaction with the seating system evaluated using the Quebec User Evaluation of Satisfaction with Assistive Technology (IT-QUEST 2.0) [26,27]) were administered to the two groups to assess their quality of life and satisfaction with the seating system; the results were compared.

The following parameters were recorded to assess any correlation with MP progression and to compare the groups:

- Slow passive ROM (pROM) in the supine position: abduction with the hip and knee flexed at 90° and the hip and knee extended (gracilis), knee extension, and hip extension according to the Thomas test;
- Pelvic tilt measured by means of degrees on the antero-posterior radiograph, acquired in the supine position, with the legs parallel to each other and the vertical line;
- Total number of botulinum (BoNT-A) injections in the hip muscles over the treatment period;
- Seating system costs for the Italian National Health System;
- Hip pain, as a dichotomous variable (pain/no pain), reported by patients or caregivers or clinically evidenced;
- MRI lesions according to the MRI classification system (MRICS) [28];
- Concurrent spasticity/dystonia treatments such as oral medication (baclofen, etc.), intrathecal baclofen (ITB) pump, and selective dorsal rhizotomy (SDR);
- Ongoing physiotherapy;
- Regular use (at least 1 h/day 5 days/week) of standing devices;
- Sex, age, and CP subtype;
- Costs of the seating devices, as declared in the estimate of the orthopedic workshop.

Before starting recruitment, training meetings were conducted involving the principal investigators and collaborators so as to agree on passive ROM assessment and identification of the best hip abduction angle to ensure hip centering while sitting, according to Lespargot’s technique [13,21]. Furthermore, written instructions were shared. More detailed information is reported in the Supplementary Data (study protocol).

A minimum sample size of 204 hips was required according to the protocol, corresponding to 102 participants. The preliminary data presented herein include patients who completed their first- and second-year assessment

Patients could discontinue the trial at any point of their own volition. Furthermore, they stopped participating in the study in cases of intolerance to the seating system, prolonged hospitalization, or being referred to orthopedic surgery because of muscle contractures or worsening of MP over 50% [12]. In the event of dropout, a final assessment was required, including clinical examination, a pelvic radiograph, and completion of the questionnaires.

Descriptive statistics are reported for baseline demographic and clinical characteristics for the entire sample as well as for each of the two groups. The analysis was conducted at the level of individual hips.

Continuous variables are presented as the number of patients (N), mean, standard deviation (SD), minimum (min), and maximum (max) and were compared between subgroups using an unpaired Student’s *t*-test. Categorical variables are presented as frequency (N, percentage [%]) and were compared using Pearson’s chi-squared test. The progression of MP in the HCS and TS arms was compared using an unpaired *t*-test.

Additionally, a stepwise selection method multiple regression analysis was performed with MP progression as the dependent variable and various acquired clinical data as independent variables. A statistical significance level of *p* < 0.05 was used to allow for the inclusion of variables in the model. Subsequently, the best multiple linear regression model was selected, and factors that independently contributed to reducing or increasing MP progression were identified.

Statistical analysis was performed using STATA^®^ software version 17 (StataCorp. 2021. Stata Statistical Software: Release 17. StataCorp LLC.: College Station, TX, USA).

More methodological details are available in the Supplementary Data (study protocol).

## 3. Results

A total of 72 patients were enrolled from October 2020 to December 2023. Of the 55 who underwent the first-year assessment and were included in this preliminary study, 29 underwent the final assessment at 24 months.

Seven patients in the HCS group dropped out of the trial: three due to family problems (no clinical events were recorded; two families moved to other rehabilitation centers; and one family had difficulties in attending the trial center); two were referred to orthopedic surgery to release the muscles of the lower limbs, though their MP was stable at under 40%; one because their MP progressed to over 50% at T12; and one due to intolerance to the seating system.

**Figure 4:**
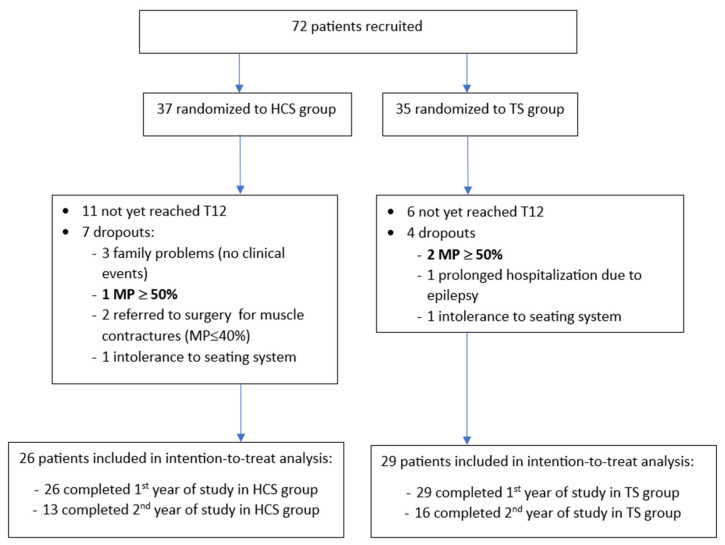
Participant flow diagram.

Four patients in the TS group dropped out of the trial: one underwent prolonged hospitalization due to severe epilepsy; two because their MP progressed to over 50% at T12; and one due to intolerance to the seating system. The participant flow diagram is presented in Figure 4.

Demographic and clinical data of the subjects are represented in Table 1. The overall mean age was 3.7 years (SD, 1.6), and 15 participants (27%) were female. The two groups were alike regarding most characteristics. Nonetheless, a significant difference was observed regarding sex (*p*-value 0.003), with a notable prevalence of males in the TS group.

Table 2 presents the characteristics of the individual hips at baseline in the overall sample and stratified by postural management approach.

The overall MP progression mean value at the 12-month assessment (T12) was 3.5 (SD 9.4), and the median value was 1.6 (IQR −2.4–8). Mean MP progression was 3.2 (SD 9.3) in the HCS group and 3.7 (SD 9.5) in the TS group. Median MP progression was 0.7 (IQR −2.2–5.7) in the HCS group and 2.2 (IQR −2.4–8.2) in the TS group. The overall MP progression mean value at the 24-month assessment (T24) was 3.9 (SD 7.9), and the median value was 2.5 (IQR −0.5–8.5). Mean MP progression was 3.6 (SD 10) in the HCS group and 4.1 (SD 5.7) in the TS group. Median MP progression was 3.3 (IQR −1.9–8.5) in the HCS group and 2.5 (IQR −0.5–8.6) in the TS group. No statistically significant difference between the TS and HCS groups was observed in terms of MP progression either at T12 or at T24. Both groups maintained mean and median MP values under 30%, with no significant difference found between the groups. Detailed results are represented in Table 3 for the overall sample and stratified by postural management approach and assessment timing (12 and 24 months).

In terms of hips exceeding MP 40%, 3.8% in the HCS group and 6.9% in TS group did so at T12, and 1.9% in the HCS group and 1.7% in the TS group did so at T24. Only 1.9% of the HCS group reached MP ≥ 50% at T12 and 5.2% in TS group; none did so at T24.

No significant changes were observed regarding pROM over time, either in the overall sample or in the individual groups.

Four hips (6.9%) were painful at T12 and one hip (2.6%) was painful at T24 in theTS group. No painful hips were reported in the HCS group at either assessment.

During the study, more BTX-A injections were administered in TS than in HCS; this was statistically significant at T12 (*p* = 0.005) but not at T24 (*p* = 0.250).

A minimum improvement was reported in both groups on the CPCHILD questionnaire, with no statistically significant difference. Similar scores were obtained for both groups on the QUEST at T12 and T24.

The cost of seating devices was significantly higher in the TS group than in the HCS group (*p* = 0.001).

A univariate linear regression analysis was performed between MP progression as the dependent variable and several independent variables. As reported in Table 4, statistically significant results were found for sex, age, CP subtype, GMFCS level, predominant grey matter injury at MRICS, drug-resistant epilepsy, and QUEST. A stepwise multiple regression analysis was then performed, and the best model included the two groups of intervention, age, and drug-resistant epilepsy. Increasing age correlated with lower MP progression values, and drug-resistant epilepsy, when present, was associated with higher MP progression values. No statistically significant association was found between MP progression and HCS or TS. Parameter estimates of the best multiple linear regression model for MP progression are reported in Table 5.

## 4. Discussion

The results at 12 months of the CP-lux-prev RCT are reported in the present study. Due to the pandemic and the fact that COVID-19 monopolized sanitary resources, dramatically reducing access to rehabilitation and medical assessments for most of chronic diseases such as CP [29], a limited sample of patients was enrolled and reached the 24-month assessment. This led to a delay to the start of the study and the recruitment of patients, which is ongoing. In any case, based on the preliminary results, indicative data emerged.

First of all, the overall sample showed lower mean MP progression compared to natural history as previously described (MP progression > 8% per year) [2], but this was also lower than previously published retrospective data from a local cohort [6]. Furthermore, this value is within the range of inter- and intra-rater reliability for the MP measure, as reported by Shore et al. [30]. Therefore, it may be assumed that the overall hip sample is stable, given the mean MP progression over the examined 12- and 24-month periods. The mean MP progression reported by Picciolini et al. [13] was 0.7% in the best hip and 1.7% in the worst hip after 1-year use of siege, but the sample included patients at GMFCS level III, who are known to present a lower risk of luxation and higher chance of spontaneous improvement. Furthermore, the study was not randomized. The present results are at odds with the findings by Kim et al. [16], which supported a negative role of adaptive seating systems with medial knee support to keep the hip mildly abducted, which caused an increase in MP compared to controls. These contrasting results are probably due to the fact that in the present study, the TS approach used adaptive postural systems that permitted adjustment based on individual characteristics and not standard wheelchairs Therefore, the pelvis and thighs were sufficiently maintained in a symmetric and stable position.

In our study protocol, we assumed the mean value of MP progression as the outcome measure to compare our data with those of previous studies, as described above. However, our results showed that MP and MP progression did not have a normal distribution in our sample; the mean values were influenced by outliers. Therefore, the median values of MP progression are more representative of the trend in our sample. Considering this, very low values of MP progression were recorded at T12 (0.6) and T24 (2.5, which represents MP progression over two years) in the entire sample. The follow-up was limited, considering the entire developmental period and considering that the risk of further displacement is still present in adulthood [4,31]. Nonetheless, two years may be considered a reasonable follow-up length to permit an assessment of the effectiveness of postural management, without resulting in the need for soft tissue surgery in this type of patient at this age. Surgery, indeed, would confound the results; its effectiveness in temporarily reducing MP is known. The novelty of the present study is that it demonstrates the role of sitting postural management, either HCS or TS, in stabilizing or slowing MP progression, but this approach must be integrated with other strategies (standing and surgery) to best manage this multifactorial clinical problem. Guidelines [7,10] agree in recommending postural management. Quadriplegic patients spend most of their time in a sitting position and need external support to maintain it for long periods of time. Offering these individuals a comfortable system that ensures axial stability and pelvis alignment is important not only in promoting wellbeing and participation but also reducing MP progression. Even a small reduction in the slope may be relevant over the course of development.

The positive role found might be attributed to the research itself, which induced increased attention to postural management and spasticity treatment when monitoring patients, in line with the evidence recommending surveillance programs [4,6,7].

An interesting result is the percentage of hips which exceeded MP 40% and MP 50%, which was lower in the HCS group. This could suggest a more protective role of HCS compared to TS. Final findings from the entire sample after two years of treatment will confirm the consistency in this trend.

Based on the regression analysis, only two factors were significantly related to MP progression: age and drug-resistant epilepsy. This is in line with results from previous studies [3,5,32]. The risk of hip luxation is higher in the first years of life, when MP increases more quickly than it does in older children. In fact, surveillance programs recommend closer clinical and radiographical assessments in these early years [7,32]. Surprisingly, no significant correlation was found between GMFCS level and MP during multiple linear regression analysis. This is at odds with previous findings [5,32,33] and will need to be verified based on our final results. Epilepsy has already been indicated among the determinants of hip displacement, in particular drug-resistant epilepsy [5,32]. It may be explained as a marker of severity, which negatively influences motor development and performance. As reported in previous studies [5,33], sex does not correlate with MP and has never been found to be a determinant of hip luxation. Therefore, the predominance of males in the TS group must not be considered relevant. The CP subtype and MRCIS did not prove to be determinants of hip luxation in these preliminary results, dissimilar to previous studies [2,5,34], but the limited sample size might have contributed to reducing the statistical impact of these factors. No association was found between MP progression and pelvic tilt; controversial results have been found previously on this topic [5,35]. In the future, more complete data will hopefully clarify these aspects. The regular use of standing devices was defined as at least one hour per day for at least five days per week. Severely affected quadriplegic patients show limited compliance with these devices in clinical practice. In fact, only 20% of our participants reported using them regularly. Dissimilar to previous studies [19], standing devices did not play any significant role in reducing MP progression in the present sample.

Quality of life (QoL) as measured using the CPCHILD questionnaire remained stable or slightly improved in both groups. Contradictory data have been published about the relationship between MP and CPCHILD scores [36]. Our sample is too limited to draw any definitive conclusions, but we can assume that postural management as proposed in the present study does not burden QoL. The extremely low incidence of hip pain is justified by the young age of the patients as it is more frequent in adolescents and adults [25,37].

No overall significant difference was observed between the groups regarding pROM. Overall values of abduction pROM corresponded to ranges previously published for patients under six years of age [38]. More BTX-A injections were administered in the TS group than in the HCS group, even though no significant difference was reported at T24. This might be related to the fact that HCS is more enveloping and adherent to the body, impeding movement inside and ensuring prolonged static stretching and benefitting tendon length [39]. On the contrary, adaptive seating systems have a reduced contact area with the body and are less effective in preventing movement of the hips and pelvis, which might elicit spastic muscles. Therefore, asymmetric positions might recur while using the system, with hyperactive muscles overcoming the antagonists.

Both groups expressed good levels of satisfaction with the seating system. This has never been enquired before. The cost of TS proved to be significantly higher than that of HCS. This is probably due to the fact that only half of the sample reached the final assessment. Data must be verified based on the final results, when the entire population will have reached the 24-month assessment. We expect the cost of HCS to increase. The TS group used adaptive systems which were more expensive but could be adjusted according to the patient’s growth, without incurring any new costs. Conversely, the HCS device was custom-made, meaning that there was limited possibility for adjustment and thus the need for a new one as the patient grew. This would imply new costs in the second year of the trial for the HCS group. Of note, costs were covered by the local health authority not the families.

### Limitations

The presented data are only preliminary. More complete results are expected at the end of the RCT.

Acetabular dysplasia, which might influence MP progression, was not measured as a secondary outcome. This could be integrated into the final study.

Only regular use (at least one hour/day for at least five days/week) was recorded, but no information was reported regarding the degree of abduction in the standing frames.

Guidelines [7,10] recommend 24 h postural management, although child compliance must be considered. As usual clinical practice, the parents were required to put a cushion between their children’s thighs at night, but in many cases, the parents reported that it was not tolerated or moved away while sleeping. The regular use of postural management devices during the night was not recorded in this study.

## 5. Conclusions

This is the first RCT comparing two sitting postural management approaches. The results are preliminary but show a general positive effect of sitting postural management in reducing MP progression.

Both HCS and TS were well tolerated, and their use did not negatively influence QoL.

## Figures and Tables

**Table 1:** Characteristics of the overall patient sample and stratified by postural management approach.

Characteristics	Total, *n* = 55	TS, *n* = 29 (52.7)	HCS, *n* = 26 (47.3)	*p*-Value
Age ^a^	3.7 ± 1.6 (1–6.9)	4.0 ± 1.6 (1.5–6.9)	3.4 ± 1.6 (1–6.9)	0.212
Sex ^b^				
Female	15 (27.3)	3 (10.3)	12 (46.2)	0.003
Male	40 (72.7)	26 (89.7)	14 (53.8)	
MRICS ^b^				
Maldevelopment	7 (12.7)	4 (13.8)	3 (11.5)	0.898
Predominant white matter injury	27 (49.1)	13 (44.8)	14 (53.9)	
Predominant grey matter injury	8 (14.6)	5 (17.3)	3 (11.5)	
Miscellaneous	13 (23.6)	7 (24.1)	6 (23.1)	
CP subtype ^b^				
Dystonic	15 (27.3)	9 (31.0)	6 (23.1)	0.480
Spastic	39 (70.9)	19 (65.5)	20 (76.9)	
Choreoathetosis	1 (1.8)	1 (3.5)	0 (0.0)	
GMFCS ^b^				
IV	29 (52.7)	14 (48.3)	15 (57.7)	0.485
V	26 (47.3)	15 (51.7)	11 (42.3)	
Drug-resistant epilepsy ^b^				
No	47 (85.5)	24 (82.8)	23 (88.5)	0.549
Yes	8 (14.5)	5 (17.2)	3 (11.5)	
Antidystonic/antispastic drugs ^b^				
No	49 (89.1)	25 (86.2)	24 (92.3)	0.469
Yes	6 (10.9)	4 (13.8)	2 (7.7)	
ITB ^b^	0	0	0	-
CPCHILD ^c^	55.7 ± 12.7 (23.7–83.8)	53.7 ± 13.4 (23.7–83.8)	57.9 ± 11.6 (32.3–78.0)	0.220

Legend: ^a^ mean value (%) ± SD (range); ^b^ n (%); ^c^ mean test scores ± SD (range).

**Table 2:** Characteristics of the individual hips at baseline in the overall sample and stratified by postural management approach.

Characteristics of the Individual Hips	Total Hips, *n* = 110	TS, *n* = 58 (52.7)	HCS, *n* = 52 (47.3)	*p*-Value
MP mean value ^a^	24.8 ± 8.5 (0–39.5)	24.8 ± 8.2 (0–39.5)	24.8 ± 8.9 (0–39)	0.989
MP median value ^b^	25.0 (19.1–31.4)	24.2 (20–31.1)	26.2 (18.9–32.6)	0.574
Pelvic tilt ^c^	0 ± 3.5 (−11–11)	0 ± 3.7 (−11–11)	0 ± 3.3 (−8–8)	1.000
pROM hip abd ^c^	48.5 ± 16.2 (10–90)	45.7 ± 18.3 (10–90)	51.6 ± 12.9 (20–80)	0.054
pROM gracilis ^c^	27.2 ± 12.3 (10–70)	25.2 ± 12.3 (10–70)	29.4 ± 12.1 (10–60)	0.073
pROM knee ext ^c^	−4.5 ± 6.6 (−30–0)	−5.0 ± 7.8 (−30–0)	−4.0 ± 4.8 (−15–0)	0.420
pROM Thomas ^c^	1.9 ± 4.4 (0–20)	2.5 ± 5.3 (0–20)	1.1 ± 3.1 (0–15)	0.091

Legend: ^a^ mean value (%) ± SD (range); ^b^ median value (%) (IQR); ^c^ mean value (degrees) ± SD (range).

**Table 3:** Outcomes for the overall sample and stratified by postural management approach.

Outcomes	Total Hips, *n* = 110				TS, *n* = 58 (52.7)				HCS, *n* = 52 (47.3)				*p*-Value, TS vs. HCS
	T0	T12	T24	*p*-Value	T0	T12	T24	*p*-Value	T0	T12	T24	*p*-Value	T0; T12; T24
MP progression Mean value ^a^	-	3.5 ± 9.4 (−13–42.7)	3.9 ± 7.9 (−1–34.7)	0.788	-	3.7 ± 9.5 (−11.3–42.7)	4.1 ± 5.7 (−8.0–14)	0.850	-	3.2 ± 9.3 (−13–32.5)	3.6 ± 10.0 (−12–34.7)	0.838	0.778; 0.852
MP progression Median value ^b^	-	1.6 (−2.6–8.0)	2.5 (−0.5–8.5)	0.264	-	2.2 (−2.4–8.2)	2.5 (−0.5–8.6)	0.878	-	0.7 (−2.2–5.7)	3.3 (−1.9–8.5)	0.241	0.230; 1.000
MP°, mean value ^a^	24.8 ± 8.5 (0–39.5)	27.8 ± 10.4 (6–77.7)	26.4 ± 7.1 (11.6–44.7)	0.052	24.8 ± 8.2 (0–39.5)	28.5 ± 11.4 (6–77.7)	27.7 ± 6.4 (16.5–44.7)	0.087	24.8 ± 8.9 (0–39)	26.8 ± 8.9 (9.1–55.2)	24.9 ± 7.7 (11.6–40.5)	0.465	0.989; 0.435; 0.159
MP, median value ^b^	25.0 (19.1–31.4)	26.0 (21.5–33.1)	26.5 (21.5–31.6)	*	24.2 (20–31.1)	25.8 (22–35.8)	27.8 (23.3–32)	°	26.2 (18.9–32.6)	26.0 (21.5–32.5)	23.8 (20.8–30)	£	0.574; 0.956; 0.108
Hip MP > 40 ^c^	0 (0.0)	6 (5.4)	2 (1.8)	0.041	0 (0.0)	4 (6.9)	1 (1.7)	0.124	0 (0.0)	2 (3.8)	1 (1.9)	0.314	-; 0.617; 0.918
Hip MP ≥ 50 ^c^	0 (0.0)	4 (3.6)	0 (0.0)	0.037	0 (0.0)	3 (5.2)	0 (0.0)	0.098	0 (0.0)	1 (1.9)	0 (0.0)	0.409	-; 0.455; -
Painful hip ^c^	2 (1.8)	4 (3.6)	1 (1.5)	0.492	2 (3.4)	4 (6,9)	1 (2.6)	0.543	0 (0.0)	0 (0.0)	0 (0.0)	-	0.177; 0.082; 0.381
Pelvic tilt ^d^	0.0 ± 3.5 (−11–11)	0.1 ± 3.3 (−10–10)	0.0 ± 3.1 (−7–7)	0.986	0.0 ± 3.7 (−11–11)	0.0 ± 3.3 (−10–10)	0.0 ± 2.9 (−7–7)	1.000	0.0 ± 3.3 (−8–8)	0.2 ± 3.3 (−6.5–7)	0.0 ± 3.4 (−7–7)	0.968	1.000; 0.811; 1.000
pROM hip abd ^d^	48.5 ± 16.2 (10–90)	49.5 ± 15.7 (10–90)	47.1 ± 10.8 (25–70)	0.623	45.7 ± 18.3 (10–90)	47.1 ± 17.7 (10–90)	46.8 ± 11.6 (25–70)	0.897	51.6 ± 12.9 (20–80)	52.8 ± 11.8 (30–80)	47.4 ± 10.1 (27–60)	0.180	0.054; 0.071; 0.852
pROM gracilis ^d^	27.2 ± 12.3 (10–70)	27.2 ± 11.9 (10–70)	25.2 ± 15.1 (10–70)	0.596	25.2 ± 12.3 (10–70)	25.5 ± 11.8 (10–70)	22.8 ± 15.0 (10–70)	0.603	29.4 ± 12.1 (10–60)	29.6 ± 11.8 (10–60)	28.3 ± 14.9 (10–55)	0.908	0.073; 0.092; 0.173
pROM knee ext ^d^	−4.5 ± 6.6 (−30–0)	−5.2 ± 7.1 (−30–0)	−3.1 ± 6.0 (−20–0)	0.063	−5.0 ± 7.8 (−30–0)	−6.5 ± 7.9 (−30–0)	−3.8 ± 6.6 (−20–0)	0.145	−4.0 ± 4.8 (−15–0)	−3.6 ± 5.5 (−20–0)	−2.3 ± 5.2 (−20–0)	0.240	0.420; 0.029; 0.200
pROM Thomas ^d^	1.9 ± 4.4 (0–20)	2.7 ± 6.6 (0–30)	2.6 ± 5.6 (0–20)	0.528	2.5 ± 5.3 (0–20)	4.3 ± 8.3 (0–30)	3.0 ± 5.6 (0–20)	0.332	1.1 ± 3.1 (0–15)	0.4 ± 1.2 (0–5)	2.1 ± 5.7 (0–20)	0.126	0.091; 0.002; 0.563
CPCHILD ^e^	55.7 ± 12.6 (23.7–83.8)	57.0 ± 10.7 (35.4–79.2)	59.1 ± 11.1 (37.9–85.1)	0.200	53.7 ± 13.3 (23.7–83.8)	56.1 ± 12.0 (35.4–77.5)	58.4 ± 11.9 (44.7–85.1)	0.220	57.9 ± 11.5 (32.3–78.0)	58.2 ± 8.6 (42.7–79.2)	59.8 ± 10.3 (37.9–73.3)	0.720	0.079; 0.325; 0.627
QUEST ^e^	-	4.1 ± 0. (2.0–5.0)	3.8 ± 0.7 (2.4–5.0)	0.057	-	4.1 ± 0.7 (2.0–5.0)	3.9 ± 0.7 (2.4–5.0)	0.583	-	4.2 ± 0.6 (2.8–5.0)	3.7 ± 0.7 (2.5–4.7)	0.022	-; 0.516; 0.301
Physiotherapy ^c^	106 (96.4)	95 (86.4)	50 (45.4)	0.281	-	54 (93.1)	26 (44.8)	0.319	-	41 (78.8)	24 (46.1)	0.600	-; 0.637; 0.496
BTX-A injections per hip	48	95	147	<0.001	33	74	97	<0.001	15	21	50	<0.001	0.123; 0.005; 0.250
Regular use of a stander ^c^	58 (52.7)	52 (47.8)	34 (30.9)	0.459		30 (51.7)	22 (37.9)	0.139		22 (42.3)	12 (23.1)	0.618	-; 0.980; 0.082
Sitting system costs ^f^	4197.6 ± 1777.2 (731.0–9069.4)				4901.0 ± 1558.8 (2963.9–9069.4)				3413.1 ± 1686.6 (731.0–6580.8)				-; -; <0.001

Legend: ^a^ mean value (%) ± SD (range); ^b^ median value (IQR); ^c^ n (%); ^d^ mean value (degrees) ± SD (range); ^e^ mean test scores ± SD (range); ^f^ mean costs (euros) ± SD (range); * statistically significant. ° between T0 vs. T12. * MP, median value ^b^ T0 vs. T12 *p* = 0.783, T0 vs. T24 *p* = 0.743, T12 vs. T24 *p* = 0.743, 0.652. ° TS-MP, median value ^b^ T0 vs. T12 *p* = 0.458, T0 vs. T24 *p* = 0.133, T12 vs. T24 *p* = 0.743, 0.546. £ HCS-MP, median value ^b^ T0 vs. T12 *p* = 0.824, T0 vs. T24 *p* = 0.423, T12 vs. T24 *p* = 0.743, 0.322.

**Table 4:** Parameter estimates of the linear regression analysis with MP progression as the dependent variable and the examined independent variables.

	Univariate Analysis			
	MP Progression T0-T12		MP Progression T0-T24	
Characteristics	B (95% CI)	*p*-Value	B (95% CI)	*p*-Value
Arm				
HCS	ref.		ref.	
TS	0.53 (−3.2–4.2)	0.778	0.40 (−3.9–4.70)	0.852
Age	−2.38 (−3.44–(−1.32))	<0.001	−2.65 (−3.71–(−1.58))	<0.001
Sex				
Female	ref.		ref.	
Male	−1.51 (−5.85–2.83)	0.490	−4.32 (−9.13–0.48)	0.077
MRICS				
Maldevelopment	ref.		ref.	
Predominant white matter injury	−6.44 (−12.31–(−0.58))	0.032	1.86 (−5.57–9.30)	0.617
Predominant grey matter injury	−3.30 (−10.27–3.66)	0.349	12.33 (4.03–20.63)	0.004
Miscellaneous	−7.45 (−13.90–(−1.00))	0.024	2.59 (−5.70–10.89)	0.553
CP subtype				
Dystonic	ref.		ref.	
Spastic	−2.85 (−16.94–1.24)	0.177	−5.46 (−10.25–(−0.66)	0.026
Choreoathetosis	−12.01 (−25.46–1.45)	0.080	−6.54 (−18.21–5.13)	0.266
GMFCS				
IV	ref.		ref.	
V	5.32 (1.78–8.87)	0.004	5.44 (1.37–9.52)	0.010
Drug-resistant epilepsy				
No	ref.		ref.	
Yes	9.12 (4.06–14.18)	0.001	13.69 (6.24–21.14)	0.001
Antidystonic/antispastic drugs				
No	ref.			
Yes	−0.87 (−6.61–4.85)	0.762	4.92 (−1.06–10.90)	0.105
ITB	-	-	-	-
CPCHILD	−0.04 (−0.22–0.13)	0.616	−0.15 (−0.34–0.03)	0.104
Quest	1.80 (−0.88–4.48)	0.186	−2.74 (−5.51–0.02)	0.052
Pelvic tilt	0.24 (0.32–0.81)	0.398	0.03 (−0.66–0.72)	0.930
pROM hip abd	−0.07 (−0.19–0.04)	0.244	0.03 (−0.18–0.24)	0.770
pROM gracilis	0.05 (−0.10–0.21)	0.473	−0.07 (−0.24–0.09)	0.357
pROM knee ext	0.12 (−0.13–0.38)	0.339	0.24 (−0.04–0.53)	0.090
pROM Thomas	0.05 (−0.23–0.33)	0.721	−0.27 (−0.66–0.12)	0.172
Hip pain	−0.3 (−9.92–9.28)	0.948	-	-
BTX-A injections	−0.05 (−1.27–1.16)	0.930	0.38 (−0.43–1.21)	0.346
Regular use of a stander	3.10 (−0.63–6.85)	0.103	1.12 (−3.28–5.54)	0.611

**Table 5:** Parameter estimates of the best multiple linear regression models for MP progression.

	Multivariate Analysis			
	MP Progression T0-T12		MP Progression T0-T24	
Characteristics	B (95% CI)	*p*-Value	B (95% CI)	*p*-Value
Arm				
HCS	ref.		ref.	
TS	1.51 (−1.89–4.91)	0.381	2.28 (−0.72–6.37)	0.116
Age	−2.23 (−3.30–(−1.15))	<0.001	−2.23 (−3.30–(−1.15))	<0.001
Drug-resistant epilepsy				
No	ref.		ref.	
Yes	7.13 (2.28–11.98)	0.004	7.64 (0.48–14.79)	0.037

## Data Availability

The raw data supporting the conclusions of this article will be made available by the authors upon request.

## Supplementary Materials

The following supporting information can be downloaded at: https://www.mdpi.com/article/10.3390/jcm13113129/s1. Supplementary Data (study protocol).
