# Supplementary material for: Sitting Postural Management to Prevent Migration Percentage Progression in Non-Ambulatory Children with Cerebral Palsy: Randomized Controlled Trial Preliminary Data
Source: J Clin Med. 2024 May 27;13(11):3129. doi: 10.3390/jcm13113129 (PMC11173266; doi:10.3390/jcm13113129)
Supplement: Supplementary file 1 [file jcm-13-03129-s001.zip › jcm-3009402-supplementary.pdf]

Sitting postural management to prevent migration percentage progression in non-ambulatory children with cerebral palsy: randomized controlled trial preliminary data

CP-lux-prev

STUDY PROTOCOL

Study setting

This is a multicenter randomized controlled trial. Thirteen Italian sites are involved: the list may be obtained consulting ClinicalTrial.gov register (NCT04603625). Study setting is outpatient, with two active arms.

Interventions

The experimental group (hip centering sitting, HCS) is intended to be sitting with an abduction-flexion angle at the hips, ensuring centering of the femoral head. This position is previously determined by a clinical examination, drawing up a hip diagram (HD) as described by Lespargot [1]. The HD identifies several positions with the hip centered into the acetabulum, by combining different angles of hip abduction and hip flexion. By palpating the femoral head at different points of the hip flexion and abduction range, the clinician identifies the position of coverage as the position in which the head is no further palpable. To reach this, moderate abduction is required, then patients in HCS group will be sitting with the hips evidently more abducted than traditionally. Considering that the objective is identifying a sitting position, the explored hip flexion range is around 90°. The optimal position is finally chosen considering individual clinical aspects, such as GMFCS level and dystonia or hypotonia. For example, in case of GMFCS V with dystonia, a hip flexion angle lesser than 90° is advisable, whenever tolerated by the child, to inhibit the global extension-torsion dystonic movements. Conversely, in case of GMFCS V with hypotonia a slightly more extended position might be more suitable. In both cases, the seating system should be reclined. Conversely, for most of children classified as GMFCS IV a vertical backrest is advisable, to facilitate head control and

participation. The desired sitting position is then maintained by a customized cast seating system, similar to the previously described “siege moulé”. [1-2]

The usual treatment group (traditional sitting, TS) is intended to be sitting simply in a comfortable and safe position, individually set, based on clinical characteristics as explained above, ensuring support and alignment of trunk and pelvis, without considering centering the femoral head into the acetabulum. This position is maintained using an adaptive seating system, for at least 5 hours/day.

In both group a comfortable and safe sitting position is guaranteed, and maximum attention is paid to facilitate child’s participation to daily activities. The seating system is placed on a wheelchair, stroller, or hi-low home base, according to familial and contextual needs; a tilt-in-space base is provided for more severe patients.

Clinicians will meet the patients and their care givers every 6 months. At these times patients are visited and adaptations or renewal of the seating systems are provided whenever needed, based on growth or clinical changes.

No expenses are attributed to the participants for the seating system, being comprised in the national free health care.

During the trial, no restrictions are imposed to any spasticity treatment, physical therapy or use of standing assistive devices. These parameters are recorded to assess any correlation with the MP, as secondary outcomes.

Eligibility criteria

Inclusion criteria are: spastic or dyskinetic CP, according to Surveillance of CP in Europe (SCPE) classification; quadriplegic patients at GMFCS level IV or V; age 1-6 years; MP<41% measured on a radiography acquired not more than 3 months prior to recruitment; informed consent of the parents or of the legal guardian, according to local Ethics Board. The cut off value of MP<41% is chosen in line with previous studies [3-5]. Hagglund et al 20144 assumed MP over 40% as hip displacement in need of operative treatment to prevent dislocation. Wordie et al. [5] identified MP 46% as a cut off limit beyond which recommending surgery, in a sample of subjects with CP at GMFCS levels III-IV-

V. Considering that the measurements of the same radiographs among multiple raters vary on average by <5% [6], the cut off value was reduced to 41%.

Exclusion criteria are: hip abduction passive range of motion (pROM) <30°; knee extension pROM limitation with flexion >15° in supine position; Thomas test >15°; anterior hip luxation; previous reconstructive surgery; soft tissue surgery in the last 12 months; lumbar scoliosis >20° Cobb.

The exclusion criteria based on pROM are to ensure compliance to the experimental sitting positioning.

The cut off values of pROM is identified as compatible with easy personal care and positioning, based on clinical experience. Furthermore, the exclusion criteria based on pROM is adopted considering the risk to get in need of muscle lengthening along the 2-year duration of study, which would impose a drop out. Anterior hip luxation is rare, and is not detected on the radiography by increased MP. Surgery is an exclusion criterion because it is known to influence the MP. Lumbar scoliosis induces a pelvic tilt which might reduce coverage of one hip, thus predisposing hip subluxation [7].

Outcomes

To compare data with previous studies [2,8-11] the mean Migration Percentage is chosen as primary outcome measure. In particular the mean Migration Percentage progression from baseline to follow-up is considered. The Migration Percentage is considered the gold standard to measure hip displacement. It is assessed on an anterior-posterior radiography of the pelvis, acquired in a supine position with the legs parallel, avoiding either pelvic rotation or anteversion. [6] The MP is calculated as the percentage of ossified femoral head laying lateral to Perkin's line. The Perkin's line is drawn through the lateral acetabular margin and perpendicular to Hilgenreiner's line. The latter one passes through the superior aspect of the triradiate cartilage. Severe acetabular dysplasia is not expected for MP<41%, but in case, identifying the acetabular margin might be challenging, then the peak of the "gothic arch" is considered to derive the Perkin's line [6]. The MP is measured for each hip, right and left one. A pelvic radiography is acquired at the recruitment phase as the baseline. Subsequent radiographies are required respectively 12 months (T12) and 24 months (T24) after start of treatment

(T0). Radiographic images from the entire sample are collected by the data manager and prepared for blinded assessment, by eliminating sensitive data and assigning an individual anonymous code. Two trained blinded evaluators independently measure the MP on such predisposed images, then the mean value is considered. An interrater difference of the MP <6% is accepted as measurement error according to Shore et al. [6] For higher disagreement, a third expert and blinded evaluator is involved and the mean value among the three assessments is accepted.

Several secondary outcomes are considered.

The percentage of hips which exceeded MP 40% was considered as a secondary outcome. As previously explained, eminent authors had assumed 40% as a cut-off value over which most hips need surgery [5] and considered keeping MP under 40% a satisfactory outcome. [4]

Two questionnaires are proposed to assess quality of life and satisfaction with the seating system and to compare groups:

- Quality of life according to the Caregivers Priorities & Child Health Index of Life with Disabilities (CPCHILD) [12-13]
- Compliance and satisfaction of the seating system evaluated by the Quebec User Evaluation of Satisfaction with Assistive Technology (IT-QUEST 2.0) [14-15]

The following parameters are recorded, to assess any correlation with the MP and to compare groups:

- Slow passive ROM (pROM) in supine position: abduction at hip and knee flexed at 90° and at hip and knee extended (gracilis), knee extension, hip extension according to the Thomas test;
- Pelvic tilt measured by means of degrees on the radiography;
- Galeazzi test in a modified version, with thighs kept vertical, laying an inclinometer on top of the knees to measure the "difference" between the two legs (the most luxated is expected to appear "shorter");
- Percentage of children who underwent botulinum (BoNT-A) injections in hip muscles;
- Seating system costs for the National Health System;

- Hip pain, as a dichotomous variable (pain/no pain), reported by patients or caregivers, or clinically evidenced;
- MRI lesions according to the MRI classification system (MRICS) by Surveillance of CP in Europe [16];
- Concurrent spasticity/dystonia treatments such as oral medication (baclofen, etc.), intrathecal baclofen (ITB) pump, selective dorsal rhizotomy (SDR);
- Ongoing physiotherapy;
- Use of standing devices (i.e., standing tables): regular use (at least 1 hour/day 5 days/week), sporadic or no use;
- Sex, age, CP subtype.

Before starting the recruitment, training meetings are performed involving principal investigators and collaborators, to agree on passive ROM assessment and identification of the best hip abduction angle to ensure hip centering while sitting, according to the Lespargot's technique. Furthermore, written instructions are shared. The clinical assessment is performed with the patient in a comfortable supine position. To measure abduction ROM the knees and hips are flexed, the hips abducted with the thighs maintained on a transverse plane tangent both hips: by means of an inclinometer the angle between the longitudinal axis of the thighs and the vertical line is measured as the abduction pROM. To measure the gracilis pROM the knees and hips are maintained extended as in the supine position, then the hips are abducted: a goniometer is positioned with the fulcrum at the anterior superior iliac spine (ASIC), easily identifiable, one arm is kept parallel to the longitudinal axis of the thigh and the other to the inter-ASIC line; finally, the angle between the thigh's axis and the perpendicular to the inter-ASIC line is calculated as the gracilis pROM. The knee extension is measured by means of a goniometer, with the fulcrum at the lateral femoral epicondyle and the arms along the femoral and tibial axis: in case of knee flexion contracture, the lacking range to reach complete extension (goniometer arms parallel, at 180°) is considered as pROM knee extension (preceded by a negative sign). Thomas test is performed to measure hip

extension range: both hips are previously flexed to resolve pelvis anteversion and lumbar lordosis, then the enquired leg is extended and the inclination of the thigh is measured by means of an inclinometer compared to the horizontal plane. In case of knee flexion contracture, foot and tibia are left dangling outside the supporting plane, to prevent limiting the hip extension.

Recruitment

Recruitment is implemented during regular clinical activity, at the participating sites. Experts in CP rehabilitation from the study sites are involved in enrollment and follow-up of the patients, such as physiatrists, child neurologists and physiotherapists. They verify the eligibility criteria, explain the study protocol and implications to the participants, and then they acquire the informed consent.

Randomization

After enrolment, participants are randomly assigned to the study arms. The randomization list was created by block randomization of random sizes of 2, 4 and 6, with a 1:1 allocation ratio, stratified according to participating sites to allow competitive enrolment. The statistical software R [17] was used to make the randomization list. Allocation is concealed by central randomisation, which is performed by statisticians who has no direct contact with the clinical aspects of the trial. The principal investigators from the study sites contacts by telephone the statisticians, who assigns participants to the study arms. Such statisticians belong to the Clinical Trials and Statistics Unit of Azienda USL - IRCCS of Reggio Emilia, which supports all telephone randomization procedures for studies conducted at this institute. Normally, the Unit responds from 8 a.m. to 6 p.m. on weekdays, but different times can be arranged upon request, even on holidays. A randomization log, mirroring the one kept at the participating clinical units, is kept at the Clinical Trials and Statistics Unit and can be checked upon request by the study monitors, if provided (or persons outside the study and the Clinical Trials and Statistics Unit assigned by the Scientific Directorate or the head of the Infrastructure, Research and Statistics Dept).

Blinding

Blinding either of the children and their caregivers or of the clinicians involved into the study is not possible. Only the evaluators of the primary outcome (MP) are blinded, by the anonymization of the radiographies.

Participant timeline

After randomization, the seating system must be predisposed. As soon as it is ready, the intervention phase may start (T0). Clinical follow-up is provided every six months (T0, T6, T12, T18, T24) by the local clinicians, a pelvic radiography and the questionnaires are required at 12 (T12) and 24 months (T24) follow-up. In case of discontinuing the study at any point other than T12 or T24, clinical and radiographic assessments and the questionnaires are required (Te). Table 1 represents the timeline of study procedures.

Table 1. *Timeline of study procedures.*

STUDY PROCEDURES	***TIMEPOINTS***						
	Enrolment	***T0***	***T6 6 months***	***T12 12 months***	***T18 18 months***	***T24 24 months***	***Te End off Study***
Exclusion criteria	**X**						
Inclusion criteria	**X**						
Informed consent	**X**						
Demographic and clinical data (sex, CP subtype, MRI lesion type, oral baclofen, ITB, BoNT-A injections in the last 6 months, drug resistant epilepsy)	**X**						
Randomization	**X**						
Devices prescription	**X**						

Devices assessment		**X**	**X**	**X**	**X**		
Clinical evaluation		**X**	**X**	**X**	**X**	**X**	**X**
Passive ROM evaluation		**X**	**X**	**X**	**X**	**X**	**X**
Modified Galeazzi test		**X**	**X**	**X**	**X**	**X**	**X**
Pelvic radiography	**X**			**X**		**X**	**X**
MP measurement	**X**			**X**		**X**	**X**
Pelvic tilt measurement		**X**		**X**		**X**	**X**
Hip pain recording		**X**	**X**	**X**	**X**	**X**	**X**
IT-QUEST 2.0				**X**		**X**	**X**
Assistive device costs recording				**X**		**X**	**X**
CPCHILD		**X**		**X**		**X**	**X**
Spasticity treatment (BoNT-A, ITB, oral drugs) recording		**X**	**X**	**X**	**X**	**X**	**X**
Use of standing device recording		**X**		**X**		**X**	**X**
Physiotherapy recording		**X**		**X**		**X**	**X**
Events recording		**X**	**X**	**X**	**X**	**X**	**X**

Monitoring compliance

To verify compliance and regular use of the seating systems (either HCS or TS), multiple approaches are implemented. First, the patients' compliance and alignment in the seating system is verified every 6 months by the principal investigator, at the follow-up visit. At this visit, the caregivers are interviewed about where (home and/or school) and how much the seating system is used and asked for any problem regarding it. Furthermore, at first and second-year assessment, caregivers are requested to answer the Quebec User Evaluation of Satisfaction with Assistive Technology questionnaire. [14-15] Finally, the trial is conducted in collaboration with the professionals who locally manage the patients and give feedback to the researchers. They may meet the patient for

neuromotor treatment, and they are in touch with the school staff (most patients attend school following an individual project). Whenever a reduced compliance is referred, the researchers re-assess the patient to exclude clinical problems that might induce discontinuing the trial. Then, they may provide modifications or renew the seating system, and plan adjunct therapies, such as botulinum injections or oral drugs.

Discontinuing the study

Patients may discontinue the trial at any point of their own volition. Furthermore, they stop participating in the study, in case of intolerance to the seating system, hospitalization for a period longer than 62 days, worsening of the MP over 50% or being addressed to orthopaedic surgery. In case of drop out, a final assessment is required (Te), providing clinical examination, a pelvic radiography, and the questionnaires.

Sample size

According to Consolidated Standards of Reporting Trials guidelines, [18] the estimated sample size is based on projected treatment effects on the primary outcome measure. An increase of the MP of 9.2% per year is described by Terjesen et al. [8] in the natural history of quadriplegic children. Then, the HCS group is expected to maintain the initial MP value, while the TS group is expected to worsen, increasing the MP of 18% over a two-year period. The analysis is conducted at the level of individual hips, as in previous studies. [5,9-11,19-20] The power analysis aimed to calculate the sample size indicates that, to detect an effect size of 18%, with a significance level of 5%, 80% of statistical power and 1:1 as allocation ratio, a minimum sample size of 204 hips is required, corresponding to 102 participants (such calculation was carried out with the nQueryAdvisor software, version 7.0). Estimating a possible dropout's percentage of 10%, a total sample of 112 subjects is preferable.

Data collection methods

Training meetings and written instructions are provided to the researchers to acquire clinical and radiographic parameters correctly. The Italian version of CPCHILD [13] and IT-QUEST 2.0 [15] questionnaires are supplied with instructions.

Data management and monitoring

Principal investigators are provided by a personal code to access an online platform (Smarty-WEB), for data entry. The electronic Case Report Form (e-CRF) is predisposed by the Institutional IT Service, according to the General Authorization to Process Personal Data for Scientific Research Purposes (as published in Italy's Official Journal n° 72 dated 26 March 2012). Participants are anonymized by assigning an individual code. Demographic and clinical data relative to recruitment and T0 to T24 or Te assessments must be inserted in compulsory fields. The data management of the coordinator site off-site monitors the overall e-CRForms, to verify completion of data.

Unintended effects or adverse events are promptly managed by the clinicians, to ensure safety and comfort of the children. They are recorded on the e-CRF at the assessment visit, which is provided every six months (T0 to T24).

Statistical methods

Statistical analysis will be conducted according to Intention To Treat principle for efficacy assessment and Treatment Received for safety assessment, in addition to the Per Protocol principle. Descriptive statistics were presented for baseline demographic clinical characteristics for the entire group, as well as for the patients with TS and with HCS. Continuous variables were presented as the number of patients (N), mean, standard deviation (SD), minimum (min), and maximum (max), and compared between subgroups using unpaired and paired Student's t test for two groups, while categorical variables were presented as frequency (N, percentage [%]) and compared using Pearson's chi-squared test.

Primary outcome

Being the MP progression the primary outcome, the analysis was performed based on the individual hips as in several previous studies. [5,9-11,19-20] The MP change in HCS and TS arms will be compared by means of the common two-sided *t* test, if homoskedasticity will be confirmed. In case of heteroskedasticity, assessed using the Folded F statistic-based test, Satterthwaite adjusted p-value

will be provided, together with the unadjusted one. Furthermore, the two-sided 95% confidence interval for the mean will be calculated.

Secondary outcomes

- The CPCHILD and IT-QUEST 2.0 scores will be analysed as for the Primary Outcome.
- The correlation between either the pelvic tilt or the Galeazzi test and the MP will be assessed using the Bravais-Pearson linear correlation coefficient, providing also the related two-sided 95% confidence interval. The estimate will also be subjected to statistical tests against the null hypothesis H0: $\rho = 0$. For the aforementioned inferential purposes, we will proceed to the *z* transformation, assuming the asymptotic normality for this transformation as usual.
- The chi-square test will be applied to compare, between arms, the percentage of subjects who has undergone each of the following treatments: BoNT-A injections, ITB, oral baclofen. The Relative Risk with its two-sided 95% confidence interval will be provided. The Spearman's rank correlation coefficient will be used to assess the relationship between the number of BoNT-A injections above mentioned and the MP for each individual hip. This correlation coefficient will be subjected to the same evaluations already described for the Bravais-Pearson statistic. For the Spearman coefficient, however, the *t* transformation will be used for inferential purposes instead of the *z* one.
- The chi-square test will be applied, in the same way, also to compare, between arms, the percentage of patients who complained hip pain, who reached an MP >40% or were addressed to surgery.
- The *t* test, applied in the same way as for the Primary Outcome, will be used to compare between arms the assistive devices cost.
- The *t* test, applied in the same way as for the Primary Outcome, will be used to compare MP between males and females and, separately, between CP subtypes; One-way variance analysis will be conducted to assess the relationship between MP, considered as dependent variable

and the MRI lesion type as grouping variable. A post-hoc Tukey test analysis will be performed in case of significant ANOVA F test.

- Similarly one-way variance analysis will be conducted to assess the relationship between MP, considered as dependent variable, and the use of standing devices (absent, sporadic, or regular) as grouping variable.

Each statistical test will be formally evaluated according to usual significance level of 5%. The statistical analysis will be conducted by the Clinical Trials and Statistics Unit staff of the coordinating site, using statistical software as SAS System, R or SPSS according to availability and in the versions in use at the time of the analyses.

Patient and public involvement statement

Patients and caregivers are involved at several stages of the trial, including the conduct of the trial. The management of both postural devices is entrusted to caregivers in daily life contexts. Patients and caregivers are encouraged to refer any problem or request concerning the device, to adapt it to their needs. Moreover, they both are involved in the outcome assessment, by filling in two questionnaires enquiring the quality of life and the compliance and satisfaction of the seating system. Finally, the research group intends to involve the families in the dissemination of the results.

References

1. Lespargot A. La luxation postéro-externe de la hanche chez l'enfant IMC ou polyhandicapé. Motricité cérébrale,1991;12:37-61.
2. Picciolini O, Le Métayer M, Consonni D et al. Can we prevent hip dislocation in children with cerebral palsy? Effects of postural management. Eur J Phys Rehabil Med. 2016 Oct;52(5):682-690.
3. Larnert P, Risto O, Hagglund G et al. Hip displacement in relation to age and gross motor function in children with cerebral palsy. J Child Orthop. 2014;8(2):129-34.

4. Hägglund G, Alriksson-Schmidt A, Lauge-Pedersen H et al. Prevention of dislocation of the hip in children with cerebral palsy: 20-year results of a population-based prevention programme. Bone Joint J. 2014 Nov;96-B(11):1546-52.

5. Wordie SJ, Bugler KE, Bessell PR, Robb JE, Gaston MS. Hip displacement in children with cerebral palsy. Bone Joint J. 2021 Feb;103-B(2):411-414. Doi: 10.1302/0301-620X.103B2.BJJ-2020-1528.R1.

6. Shore BJ, Martinkevich P, Riazi M, Baird E, Encisa C, Willoughby K, et al. CHOP Investigative Team. Reliability of Radiographic Assessments of the Hip in Cerebral Palsy. J Pediatr Orthop. 2019 Aug;39(7):e536-e541. Doi: 10.1097/BPO.0000000000001318.

7. Hägglund G. Association between pelvic obliquity and scoliosis, hip displacement and asymmetric hip abduction in children with cerebral palsy: a cross-sectional registry study. BMC Musculoskelet Disord. 2020 Jul 14;21(1):464. Doi: 10.1186/s12891-020-03484-y

8. Terjesen T. The natural history of hip development in cerebral palsy. Dev Med Child Neurol. 2012;54:951-7.

9. Faccioli S, Sassi S, Ferrari A, Corradini E, Toni F, Kaleci S, et al. Hip subluxation in Italian cerebral palsy children and its determinants: a retrospective cohort study. Int J Rehabil Res. 2022 Dec 1;45(4):319-328. Doi: 10.1097/MRR.0000000000000545.

10. Graham HK, Boyd R, Carlin JB, Dobson F, Lowe K, Nattrass G, et al. Does botulinum toxin a combined with bracing prevent hip displacement in children with cerebral palsy and "hips at risk"? A randomized, controlled trial. J Bone Joint Surg Am. 2008 Jan;90(1):23-33. Doi: 10.2106/JBJS.F.01416.

11. Willoughby K, Ang SG, Thomason P, Graham HK. The impact of botulinum toxin A and abduction bracing on long-term hip development in children with cerebral palsy. Dev Med Child Neurol. 2012 Aug;54(8):743-7.

12. Narayanan UG, Fehlings DL, Weir S, Knights S, Kiran S, Campbell K. Caregiver Priorities & Child Health Index of Life with Disabilities: initial development and validation of an outcome measure of health status and well-being in children with severe cerebral palsy. Dev Med Child Neurol, 2006; 48: 804-812.

13. Sproccati N, Bertana S, Battisti N, Feliciangeli A, Baroncini C, Zenesini C, Cersosimo A. Italian translation and cross-cultural adaptation of the questionnaire for the assessment of quality of life in children with cerebral palsy: caregiver priorities and child health index of life with disabilities. Minerva Med. 2019 Jun 25.

14. Demers L, Weiss-Lambrou R, Ska B. Item analysis of the Quebec User Evaluation of Satisfaction with Assistive Technology (QUEST). Assist Technol. 2000;12(2):96-105.

15. Galeoto G, Colucci M, Guarino D, Esposito G, Cosma E, De Santis R, Grifoni G, Valente D, Tofani M. Exploring Validity, Reliability, and Factor Analysis of the Quebec User Evaluation of Satisfaction with Assistive Technology in an Italian Population: A Cross-Sectional Study. Occup Ther Health Care, 2018;32(4):380-92.

16. Himmelmann K, Horber V, De La Cruz J, Horridge K, Mejaski-Bosnjak V, Hollody K, Krägeloh-Mann I; SCPE Working Group. MRI classification system (MRICS) for children with cerebral palsy: development, reliability, and recommendations. Dev Med Child Neurol. 2017 Jan;59(1):57-64.

17. R Core Team (2022). R: A language and environment for statistical computing. R Foundation for Statistical Computing, Vienna, Austria. URL https://www.R-project.org/.

18. Schulz KF, Altman DG, Moher D; CONSORT Group. CONSORT 2010 statement: updated guidelines for reporting parallel group randomised trials. BMJ. 2010 Mar 23;340:c332.

19. Faccioli S, Sassi S, Corradini E, Toni F, Kaleci S, Lombardi F, et al. A retrospective cohort study about the hip luxation in non-ambulatory cerebral palsy patients: the point of no return. J Child Orthop. 2022, Vol. 16(3) 227–232. Doi: 10.1177/18632521221106361.

20. Kim IS, Park D, Ko JY, Ryu JS. Are Seating Systems With a Medial Knee Support Really Helpful for Hip Displacement in Children With Spastic Cerebral Palsy GMFCS IV and V? Arch Phys Med Rehabil. 2019 Feb;100(2):247-253. doi: 10.1016/j.apmr.2018.07.423.
